# Circulating angiogenic factors and HIV among pregnant women in Zambia: a nested case–control study

**DOI:** 10.1186/s12884-021-03965-5

**Published:** 2021-07-28

**Authors:** Megan E. Smithmyer, Chileshe M. Mabula-Bwalya, Humphrey Mwape, Gabriel Chipili, Bridget M. Spelke, Margaret P. Kasaro, Kristina De Paris, Bellington Vwalika, Yuri V. Sebastião, Jeffrey S.A. Stringer, Joan T. Price

**Affiliations:** 1 Division of Global Women’s Health, Department of Obstetrics and Gynecology, University of North Carolina at Chapel Hill, Chapel Hill, NC USA; 2 University of North Carolina Global Projects Zambia, Lusaka, Zambia; 3 Department of Obstetrics and Gynaecology, University of Zambia School of Medicine, Lusaka, Zambia; 4 Department of Microbiology and Immunology, University of North Carolina at Chapel Hill, Chapel Hill, NC USA

**Keywords:** HIV, Placental growth factor, Soluble fms-like tyrosine kinase-1, Preterm birth, Stillbirth

## Abstract

**Background:**

Maternal HIV increases the risk of adverse birth outcomes including preterm birth, fetal growth restriction, and stillbirth, but the biological mechanism(s) underlying this increased risk are not well understood. We hypothesized that maternal HIV may lead to adverse birth outcomes through an imbalance in angiogenic factors involved in the vascular endothelial growth factor (VEGF) signaling pathway.

**Methods:**

In a case–control study nested within an ongoing cohort in Zambia, our primary outcomes were serum concentrations of VEGF-A, soluble endoglin (sEng), placental growth factor (PlGF), and soluble fms-like tyrosine kinase-1 (sFLT-1). These were measured in 57 women with HIV (cases) and 57 women without HIV (controls) before 16 gestational weeks. We used the Wilcoxon rank-sum and linear regression controlling for maternal body mass index (BMI) and parity to assess the difference in biomarker concentrations between cases and controls. We also used logistic regression to test for associations between biomarker concentration and adverse pregnancy outcomes (preeclampsia, preterm birth, small for gestational age, stillbirth, and a composite of preterm birth or stillbirth).

**Results:**

Compared to controls, women with HIV had significantly lower median concentrations of PlGF (7.6 vs 10.2 pg/mL, *p *= 0.02) and sFLT-1 (1647.9 vs 2055.6 pg/mL, *p *= 0.04), but these findings were not confirmed in adjusted analysis. PlGF concentration was lower among women who delivered preterm compared to those who delivered at term (6.7 vs 9.6 pg/mL, *p *= 0.03) and among those who experienced the composite adverse birth outcome (6.2 vs 9.8 pg/mL, *p *= 0.02). Median sFLT-1 concentration was lower among participants with the composite outcome (1621.0 vs 1945.9 pg/mL, *p *= 0.04), but the association was not significant in adjusted analysis. sEng was not associated with either adverse birth outcomes or HIV. VEGF-A was undetectable by Luminex in all specimens.

**Conclusions:**

We present preliminary findings that HIV is associated with a shift in the VEGF signaling pathway in early pregnancy, although adjusted analyses were inconclusive. We confirm an association between angiogenic biomarkers and adverse birth outcomes in our population. Larger studies are needed to further elucidate the role of HIV on placental angiogenesis and adverse birth outcomes.

**Supplementary Information:**

The online version contains supplementary material available at 10.1186/s12884-021-03965-5.

## Background

1.5 million women with HIV become pregnant every year, with 87% living in Sub-Saharan African countries, like Zambia [1]. Despite the progress that has been made in expanding access to antiretroviral therapy (ART), women with HIV are still at increased risk for preterm birth (PTB), fetal growth restriction (FGR), and stillbirth [2]. Studies of placental development in women with HIV have demonstrated alterations in the vascularization of the placenta that are associated with pregnancy complications like PTB and FGR [3, 4]. Others have shown that among women with HIV, angiogenic imbalance, which can lead to dysregulated placental vascularization, is associated with pregnancy complications and adverse birth outcomes [5, 6].

In early pregnancy, the formation and expansion of blood vessels within the placenta are promoted through the vascular endothelial growth factor (VEGF) signaling pathway, which is tightly regulated by pro-angiogenic (VEGF-A, placental growth factor [PlGF]) and anti-angiogenic (soluble fms-like tyrosine kinase -1 [sFLT-1], soluble endoglin [sEng]) signaling proteins [7, 8]. Imbalances in these proteins have been linked to pregnancy-related adverse outcomes such as miscarriage [9–11], spontaneous PTB [12], FGR [5, 12, 13], and stillbirth [5, 14]. While these studies showed that angiogenic imbalance is associated with adverse birth outcomes among women with and without HIV from distinct cohorts, it is not clear whether HIV infection itself is related to angiogenic imbalance in a single cohort. If HIV infection induces an angiogenic imbalance in pregnant women, that could provide insight into a mechanistic explanation for the increased incidence in adverse birth outcomes observed in women with HIV and could present an opportunity for future therapeutic interventions. To test the hypothesis that HIV infection is associated with angiogenic imbalance in early pregnancy, we conducted a nested case–control study comparing concentrations of angiogenic signaling proteins between women living with HIV and those without.

## Methods

### ZAPPS cohort description

This study used clinical data and biorepository specimens collected from pregnant women enrolled in the Zambian Preterm Birth Prevention Study (ZAPPS; ClinicalTrials.gov Identifier: NCT02738892). ZAPPS is an ongoing observational antenatal cohort established at the Women and Newborn Hospital of the University Teaching Hospital in Lusaka to characterize factors associated with adverse birth outcomes. The data and specimens used in the current analysis are all derived from participants in the first phase of ZAPPS, which completed enrollment in September 2017 and follow-up in June 2018 [15, 16].

ZAPPS participants are enrolled before 24 gestational weeks, as determined by ultrasound at screening. All participants are tested for HIV with the Alere Determine HIV-1/2 test kit (Abbot Diagnostics, Lake Forest, Illinois, USA). Positive results are confirmed with the SD Bioline 3.0 (SD Biostandard Diagnostics, Gurgaon, Haryana, India). Women newly diagnosed with HIV are counseled and referred to the appropriate HIV care and treatment services. In Zambia, immediate ART initiation is recommended for pregnant and breastfeeding women, with most pregnant women receiving the first-line combination of tenofovir disoproxil fumarate, emtricitabine, and efavirenz. For participants who test positive for HIV, viral load is measured at enrollment using a GeneXpert (Cepheid, Sunnyvale, California, USA).

Participants who enroll in ZAPPS are provided routine antenatal care at enrollment and monthly thereafter in line with Zambian guidelines. At delivery, research nurses collect details regarding each participant’s labor and delivery course, as well as maternal and neonatal outcomes by either direct observation/record review at the time of delivery, or at first contact with the participant postpartum [15, 16].

### Study population

We performed a nested case–control study comparing serum concentrations of VEGF-A, sEng, PlGF, and sFLT-1 between cases and controls. Cases were defined as participants with HIV at enrollment for whom a serum sample collected before 16 gestational weeks was available. Because concentrations of these protein biomarkers are known to vary over the course of pregnancy [17], controls were chosen from participants without HIV at enrollment and matched on gestational age at sample collection.

All ZAPPS participants provide individual written informed consent before undergoing study procedures. The University of Zambia Biomedical Research Ethics Committee, the National Health Research Authority of the Zambian Ministry of Health, and the University of North Carolina at Chapel Hill Institutional Review Board each approved the use of these clinical data and biological specimens for research purposes.

### Specimen collection

As part of the ZAPPS protocol, maternal serum samples are collected at the enrollment visit. Specimens are transferred from the study clinic to the UNC GPZ on-site research laboratory within two hours of specimen collection. There, specimens are processed into 1 mL aliquots and then transferred to -80 °C freezers. All serum samples used in this analysis were subjected to no more than two freeze–thaw cycles.

### Assay procedures

Concentrations of VEGF-A, sEng, PlGF, and sFLT-1 were measured in thawed serum using the Luminex MAGPIX analyzer (Merck, Darmstadt, Germany) with kits purchased from R&D Biosystems (Minneapolis, MN; catalogue # LXSAHM; Lot # L127927) at our laboratory in Lusaka. VEGF-A was run separately from sEng, PlGF, and sFLT-1 to avoid antibody cross-reactivity. Samples were processed according to manufacturer instructions. Serum was diluted in calibrator diluent at a 1:3 ratio. A minimum of 50 microparticles were captured for each analyte.

### Statistical analysis

Descriptive statistics were collected as part of the ZAPPS protocol at the first antenatal visit. We reported these statistics for cases and controls as n (%) and median (interquartile range). We used Wilcoxon rank sum test to compare continuous variables and the χ^2^ test to compare categorical variables between cases and gestational age-matched controls.

Biomarker concentrations were reported as medians and interquartile ranges (IQR) for cases and controls. In unadjusted analysis, we used the non-parametric Wilcoxon rank-sum test to assess differences in biomarker concentration between cases and controls. In adjusted analysis, we constructed a linear regression model for each biomarker using log-transformed biomarker concentration as the dependent variable and HIV status as the independent variable, adjusting for potential confounders based on our demographic data and variables that have the potential to be confounding based on reports in the literature. The PlGF/sFLT-1 ratio has been cited as a useful predictor of placental dysfunction [18]. To see if this ratio differed among cases and controls we build a linear regression model with log-transformed PlGF/sFLT-1 as the dependent variable and HIV status as the independent variable. We also conducted an adjusted analysis, adjusting for potential confounders as described above.

Among participants with HIV, we conducted unadjusted analyses to quantify the association of angiogenic biomarker concentration by viral load (i.e., detectable vs. undetectable) at enrollment and by timing of ART initiation (preconceptional vs. not preconceptional). For these analyses we used the Wilcoxon rank-sum non-parametric test of association.

We built unadjusted and adjusted logistic regression models to investigate the relationship between biomarker concentration and adverse outcomes in our cohort. Preeclampsia was defined as both elevated blood pressure (≥ 140 mmHg systolic or ≥ 90 mmHg diastolic) and proteinuria (≥ 2 protein on urine dipstick). PTB was defined as delivery between 16 and 36 ^6^/_7_ gestational weeks. The lower limit of 16 weeks was chosen based on evidence that the mechanisms underlying preterm birth at 16 weeks do not substantially differ from the mechanisms underlying preterm birth after 20 weeks [19, 20]. Among participants who experienced a PTB in this nested study, the median gestational age at delivery was 31.9 weeks (IQR 20.6 – 35.3 weeks). As angiogenic imbalance could contribute to both spontaneous (e.g., spontaneous labor onset in the setting of placental abruption) and provider-initiated preterm birth phenotypes (e.g., induction of labor for preeclampsia), we did not differentiate between spontaneous PTB and provider-initiated PTB in case selection. We used small-for-gestational age infant (SGA) as a proxy for FGR and defined SGA as birth weight below the 10^th^ percentile for gestational age based on INTERGROWTH-21^st^ standards [21]. Stillbirth was defined as infants born beyond 16 weeks gestational age without signs of life. Finally, we also evaluated a composite adverse birth outcome of PTB and/or stillbirth at any gestational age. In adjusted analysis, we adjusted for potential confounders based on our demographic data and variables that have the potential to be confounding based on prior reports. In all cases, biomarkers were converted to deciles before inclusion as the independent variable in the logistic regression model so that the odds ratio represents the odds of the adverse event of interest associated with a one decile increase in biomarker concentration.

The sample size for this study was driven by the availability of eligible samples in the Lusaka biorepository. All samples that met the eligibility criteria for cases (collected from women with HIV at the appropriate gestational age) were included in the study. These cases were matched based on gestational age with controls at a ratio of 1 case to 1 control. All statistical analyses were performed using STATA (version 15.1; StataCorp LP, College Station, TX). Statistical significance was set at a threshold of *p *< 0.05.

## Results

### Study population

**Fig. 1:**
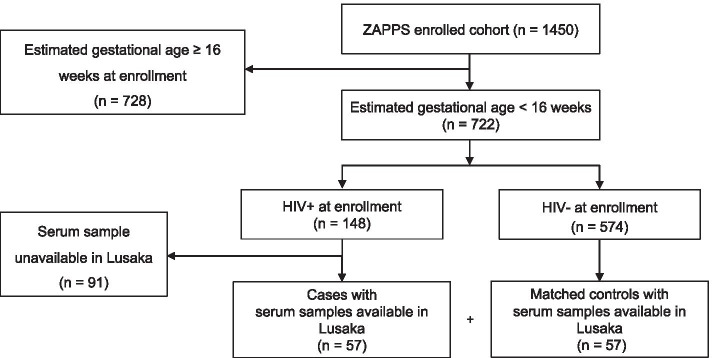
Sample inclusion criteria flowchart. This flowchart describes the criteria used to select the subset of ZAPPS specimens that were included in this analysis as either cases or controls

Between August 2015 and September 2017, 1450 participants were enrolled in ZAPPS. This cohort included 722 (49.8%) women who enrolled prior to 16 weeks gestational age, the target population for this research (Fig. 1). Of those 722 women, 148 had HIV. Of those, 91 participants did not have a sample available, either because the sample had been used for other testing or because the sample had been shipped to our sister laboratory at the University of North Carolina at Chapel Hill. We analyzed all 57 cases with eligible samples remaining in Lusaka and selected 57 gestational age-matched controls among uninfected women with eligible serum samples available. The demographic variables of the subset of participants included in this study were compared to the overall ZAPPS cohort and were not found to differ substantially (Additional file 1).

### Baseline characteristics

Participants with HIV were modestly older (median age: 29 vs. 27 years, *p *= 0.2) and had higher parity (median prior deliveries: 2 vs. 1, *p *= 0.001) than participants without HIV (Table 1). Participants with HIV had modestly lower BMI’s (median BMI: 23.87 vs 25.83 kg/m^2^*p *= 0.2). The frequencies of prior PTB and prior stillbirth were modestly higher among participants with HIV (*p *= 0.09 and *p *= 0.2, respectively).

**Table 1:** Baseline characteristics

**Characteristic**	**N**	**HIV-**	**N**	**HIV + **	p-value*
		**% or** **Median (IQR)**		**% or** **Median (IQR)**	
Age, years	53	27 (24—31)	57	29 (24—34)	0.20
< 20	2	3.77	2	3.51	0.95
20–34	42	79.25	44	77.19	
≥ 35	9	16.98	11	19.30	
Missing	4		0		
Marital status					> 0.99
Not married and not cohabiting	6	10.53	6	10.53	
Married or cohabiting	51	89.47	51	89.47	
Missing	0		0		
BMI, kg/m2	56	25.83 (21.09—29.57)	55	23.87 (21.45—28.32)	0.20
< 18.5	3	5.36	5	9.09	0.64
18.5—30.0	40	71.43	40	72.73	
> 30	13	23.21	10	18.18	
Missing	1		2		
Gestational age at specimen collection, weeks	57	12.71 (10.71—14.86)	57	12.57 (10.43—14.86)	0.90
< 14, weeks	36	63.16	36	63.16	1.00
≥ 14	21	36.84	21	36.84	
Parity	57	1 (0—2)	57	2 (1—3)	> 0.01
Nulliparous	23	40.35	5	8.77	> 0.01
Parous	34	59.65	52	91.23	
Prior PTB among parous, n = 86					
no prior PTB	14	41.18	31	59.62	0.09
≥ 1 prior PTB	20	58.82	21	40.38	
Prior stillbirth among parous, n = 86					
no prior SB	24	72.73	41	85.42	0.20
≥ 1 prior SB	9	27.27	7	14.58	
Missing	1		4		
Blood pressure at enrollment					
Normotensive	53	94.64	52	92.86	0.70
Hypertensive	3	5.36	4	7.14	
Missing	1		1		
Syphilis at enrollment					
Non-reactive	53	94.64	49	87.50	0.20
Reactive	3	5.36	7	12.50	
Missing	1		1		
Hemoglobin at enrollment, mg/dL	47	12 (12—13)	45	12 (11—13)	0.50
≥ 11	45	95.74	38	84.44	0.07
< 11	2	4.26	7	15.56	
Missing	10		12		
Malaria at enrollment					
Negative	41	100.00	49	98.00	0.40
Positive	0	0.00	1	2.00	
Missing	16		7		
Urinalysis at enrollment					
Normal	52	92.86	51	89.47	0.50
Abnormal^a^	4	7.14	6	10.53	
Missing	1		0		

^*^ p-value calculated using Wilcoxon ranksum test for continuous variables and chi-square for categorical variables

^a^ Defined as 1 + leukocyte esterase and/or + nitrates

### Association between HIV and angiogenic biomarkers

VEGF-A concentrations were below the limit of detectability of the Luminex assay in all samples, a limitation previously observed in other antenatal cohorts, which may be the result of increased circulation of VEGF-A binding proteins like sFLT-1 during pregnancy [5, 22]. Since the concentrations of sEng, PlGF, and sFLT-1 were not normally distributed, the non-parametric Wilcoxon rank-sum test was used to test for differences between cases and controls in unadjusted analysis.

**Fig. 2:**
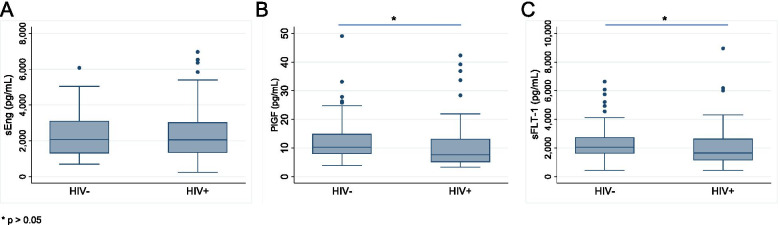
Concentrations of **A**) sEng **B**) PlGF and **C**) sFLT-1 in 57 controls and 57 cases, analyzed using Luminex assays. The threshold for statistical significance was set to *p *< 0.05, as calculated using Wilcoxon rank-sum

sEng concentration was similar between cases with HIV (median 2055.4 pg/mL, IQR: 1340.7 – 3026.5) and controls without HIV (median 2061.5 pg/mL, IQR: 1306.4 – 3100.3; *p *= 0.96). PlGF concentration was lower among women with HIV (median 7.6 pg/mL, IQR: 5.1 – 13.2) than those without HIV (median 10.2 pg/mL, IQR: 7.9 – 14.9; *p *= 0.02) (Fig. 2). sFLT-1 concentration was also lower in cases (median 1647.9 pg/mL, IQR: 1140.4 – 2641.8) than in controls (median 2055.6 pg/mL, IQR: 1619.7 – 2735.2; *p *= 0.04).

We constructed linear regression models of the association between each log-transformed biomarker, the log-transformed ratio of PlGF/sFLT-1 which has been proposed as an early marker of placental dysfunction [18], and maternal HIV serostatus. We adjusted this model for maternal BMI based on previous reports that elevated BMI is associated with increased concentrations of angiogenic factors [23]. We also adjusted for parity to account for the difference in parity observed between cases and controls. In this analysis, we found concentrations of PlGF and sFLT-1 were not independently associated with HIV (Table 2).

**Table 2:** Association between angiogenic biomarker concentrations among 57 participants with HIV compared to 57 without HIV (n = 57) in ZAPPS

	**HIV-**	**HIV + **	**β**	**95% CI**	***p-value***	β*	95% CI*	p-value*
	**Median (IQR)**	**Median (IQR)**						
sEng	2061.51 (1306.36 – 3100.25)	2055.37 (1340.71 – 3026.46)	0.002	-0.22—0.22	0.99	0.01	-0.23—0.26	0.92
PlGF	10.24 (7.91 – 14.87)	7.63 (5.07 – 13.15)	-0.23	-0.46—0.00	0.06	-0.20	-0.46—0.05	0.12
sFLT-1	2055.63 (1619.70 – 2735.15)	1647.88 (1140.42 – 2641.84)	-0.22	-0.45—0.00	0.05	-0.06	-0.30—0.18	0.63
PlGF/sFLT-1	0.005 (0.003 – 0.009)	0.005 (0.003 – 0.008)	-0.004	-0.30 – 0.29	0.98	-0.14	-0.47 – 0.18	0.38

β, 95% CI, and *p-*values calculated by linear regression of log-transformed biomarker concentration, pg/mL

^*^adjusted for maternal BMI and parity

Of the 57 cases included in this sub-study, 19 (33.3%) had a detectable viral load at enrollment. Detectable viral load at enrollment was not associated with differences in biomarker concentrations in unadjusted analyses (Additional file 2). Similarly, biomarker concentrations did not differ between the 40 (70.2%) women with HIV who had initiated ART prior to conception compared to the 17 (29.8%) who had not (Additional file 3).

### Angiogenic biomarkers and adverse outcomes

Thirty participants (21 cases, 9 controls) in this study experienced an adverse outcome. The proportions of adverse outcomes across cases and controls were as follows: 4 preeclampsia (2 cases, 2 controls), 11 PTB (9 cases, 2 controls), 17 SGA (12 cases, 5 controls), 5 stillbirth (5 cases, 0 controls), 12 composite adverse birth outcome (10 cases, 2 controls). In unadjusted analysis using logistic regression, participants who experienced a PTB had lower concentrations of PlGF than participants who delivered at term (0.71, 95% CI: 0.54 – 0.93; *p *= 0.01) (Table 3). PlGF concentrations were also lower among participants with a stillbirth compared to participants who delivered a liveborn infant (0.64, 95% CI: 0.41 – 1.01; *p *= 0.05), and participants with the composite adverse outcome variable compared to women who delivered a liveborn infant at term (0.68, 95% CI: 0.52 – 0.90; *p *= 0.01). Participants who experienced the composite adverse birth outcome also had lower concentrations of sFLT-1 compared to those who delivered a liveborn infant at term (0.78, 95% CI: 0.61 – 0.99; *p *= 0.04). sEng concentration was not associated with any adverse outcomes.

**Table 3:** Associations between adverse outcomes and biomarker concentrations

	**Odds Ratio**^**ǂ**^	**95% CI**	**adjusted Odds Ratio**^**ǂ**^**^**	**adjusted 95% CI^**
Preeclampsia (n = 4, missing = 59)				
sEng	1.02	0.71 – 1.45	1.06	0.70 – 1.60
PlGF	1.00	0.68 – 1.45	1.02	0.68 – 1.53
sFLT-1	1.20	0.81 – 1.77	1.33	0.86 – 2.05
Preterm Birth (n = 11, missing = 17)				
sEng	0.96	0.77 – 1.19	1.00	0.79 – 1.27
PlGF	0.71	0.54 – 0.93	0.73	0.55 – 0.97
sFLT-1	0.81	0.64 – 1.03	0.89	0.69 – 1.15
SGA (n = 17, missing = 24)				
sEng	1.04	0.87 – 1.26	0.99	0.80 – 1.22
PlGF	1.05	0.87 – 1.26	1.05	0.86 – 1.29
sFLT-1	0.87	0.72 – 1.05	0.92	0.74 – 1.14
Stillbirth (n = 5, missing = 19)				
sEng	0.87	0.63 – 1.20	0.93	0.64 – 1.36
PlGF	0.64	0.41 – 1.01	0.71	0.44 – 1.13
sFLT-1	0.66	0.43 – 1.02	0.78	0.49 – 1.24
Adverse Birth Outcome* (n = 12, missing = 19)				
sEng	0.92	0.75 – 1.14	0.96	0.76 – 1.21
PlGF	0.68	0.52 – 0.90	0.70	0.53 – 0.93
sFLT-1	0.78	0.61 – 0.99	0.86	0.67 – 1.12

ǂCoefficient is for one decile increase in the analyte

*Adverse birth outcome is a combined variable including stillbirth and gestational age of <37 weeks at delivery

^Adjusted for maternal BMI at enrollment, parity, and HIV status

In adjusted analysis using logistic regression, lower PlGF concentrations were associated with participants who experienced a PTB compared to those who delivered at term, adjusting for HIV status, BMI, and parity (0.73; 95% CI: 0.55 – 0.97; *p *= 0.03). Similarly, lower PlGF was associated with women who experienced either a preterm birth or stillbirth compared to those who delivered a liveborn infant at term (0.70; 95% CI: 0.53 – 0.93; *p *= 0.02). Concentrations of sFLT-1 were not associated with the composite adverse birth outcome in adjusted analysis. In all cases, odds radios should be interpreted as the odds of an adverse event associated with a one decile increase in biomarker concentration.

## Discussion

In this nested case–control study, we found that HIV was associated with significantly lower PlGF and sFLT-1, but these findings were not confirmed in adjusted analysis. This preliminary finding supports the hypothesis that HIV infection disrupts the VEGF signaling pathway that regulates angiogenesis and vasculogenesis during placental development. VEGF-A, sEng, PlGF, and sFLT-1 help regulate the vascularization of the placenta either as promoters of angiogenesis (VEGF-A and PlGF) or antagonists (sFLT-1 and sEng). Imbalances in these biomarkers during pregnancy cause alterations in placental vascularization, jeopardizing blood flow to the developing fetus and leading to PTB, FGR, and stillbirth [24]. This hypothesis aligns with reports in the literature that HIV is associated with dysregulated placental development. In particular, one study comparing placentas collected from 120 women with HIV and 103 women without HIV found that HIV was associated with increased incidence of maternal vascular malperfusion [4]. Another study found that in placentas collected from 38 women with HIV who experienced a preterm birth and 43 women without HIV who experienced a preterm birth, HIV was associated with thrombosis, infarction, anomalies in cord insertion, and hypervascularization [3]. It is noteworthy that in both studies, the women with HIV were also being treated with ART, thus the effects of HIV and ART cannot be decoupled. We did not detect differences in biomarker concentration between participants who received ART preconceptionally and those who did not, but our sample size for this analysis was small and vulnerable to Type II error. Additional studies are needed to confirm the results of this preliminary data and to better understand the relationship between ART, HIV, and VEGF signaling. This information could provide insights for future therapies to address the increased risk of adverse birth outcomes in women with HIV.

In this study, lower concentrations of PlGF were associated with preterm birth and the composite adverse birth outcome. Lower sFLT-1 concentrations were associated with the composite adverse birth outcome in unadjusted but not in adjusted analysis. We found no association between HIV and sEng concentration, nor between sEng and any adverse birth outcomes. Our findings align with reports in other populations that low PlGF and sFLT-1 concentrations are associated with adverse birth outcomes. In a UK study of 981 asymptomatic pregnant women, both PlGF and sFLT-1 levels were reduced in women who experienced a miscarriage [10]. Another study of 1876 women participating in a prospective cohort study of Down syndrome screening found that higher levels of sFLT-1 were associated with reduced risk of spontaneous PTB and stillbirth [12]. Similarly, in a study of 389 pregnant women with HIV participating in a trial of protease inhibitor vs non-nucleoside reverse transcriptase inhibitor-based ART in Uganda, lower PlGF concentrations were associated with stillbirth. [5] Although our study found no association between sEng concentration and adverse outcomes, others have found that elevated sEng is associated with miscarriage [10], PTB, and stillbirth in women with HIV [5]. Notably, both these findings were derived from longitudinal measurements of sEng throughout pregnancy. It is possible that an association between sEng and adverse outcomes could be observed in our population at later timepoints. We did not observe any association between preeclampsia and sEng, PlGF, or sFLT-1, but others have observed that the PlGF/sFLT-1 ratio could be a useful early predictor of preeclampsia [25]. However, a recent study investigating PlGF and sFLT-1 concentrations in normotensive and preeclamptic women with and without HIV in late pregnancy found that preeclampsia was not associated with a lower PlGF/sFLT-1 ratio in women with HIV [6]. One limitation of this study was the large amount of missingness in the preeclampsia outcome, making it difficult to draw conclusions based on this data. Further studies specifically focused on HIV and preeclampsia could provide better insights into this relationship.

The key strength of this study was the ability to directly compare biomarker concentrations in women with and without HIV in early pregnancy. Others have noted the need for this comparison to determine the effects of HIV infection on angiogenic imbalance and dysregulated placental development [5]. Additionally, all ZAPPS participants received early ultrasound biometry, allowing for more accurate gestational age estimation than in cohorts that rely on last menstrual period, fundal height, or infant exam [26].

The authors acknowledge that this study had several limitations. The sample size was limited by specimen availability and larger studies are needed to confirm these results. Additionally, limited power may have contributed to the lack of effect observed in adjusted analysis. This analysis was limited to a single timepoint in early pregnancy and longitudinal analysis could provide more insight into the relationship between HIV and these biomarkers over time. The authors acknowledge that the preterm birth outcome included both spontaneous preterm birth and provider initiated preterm birth, which could both be affected by angiogenic imbalance but would likely have different underlying mechanisms. A larger sample size would allow for these two outcomes to be assessed individually. Finally, this study was limited to Zambian women living in the Lusaka urban area and may not be generalizable to other populations.

## Conclusions

This study found preliminary evidence that pregnant women with HIV may have lower levels of circulating PlGF and sFLT-1 in early pregnancy compared to women without HIV, although our findings were not conclusive in adjusted analyses. We confirmed previous findings that lower concentrations of PlGF are associated with an increased prevalence of preterm birth and the composite adverse birth outcome (preterm birth or stillbirth) in our population. Future studies with a larger sample size are needed to confirm the association or lack of association between HIV and dysregulated angiogenesis. Further insights into the mechanism by which HIV increases the risk of adverse outcomes could aid in the development of therapeutics to address this risk and improve outcomes for millions of women living with HIV.

## Supplementary Information

**Additional file 1.** A table comparing demographic variables between the overall ZAPPS cohort and this nested case-control study.

**Additional file 2.** A table describing the relationship between detectable viral load and angiogenic biomarker concentration.

**Additional file 3.** A table describing the relationship between ART timing and angiogenic biomarker concentration.

## Data Availability

The dataset used in this study is available to the public as part of the OSF database (link: https://osf.io/f2tzs/).

## Abbreviations

ART: Antiretroviral therapy
BMI: Body mass index
FGR: Fetal growth restriction
IQR: Interquartile range
PlGF: Placental growth factor
PTB: Preterm birth
sENG: Soluble endoglin
SGA: Small for gestational age
sFLT-1: Soluble fms-like tyrosine kinase 1
VEGF: Vascular endothelial growth factor
ZAPPS: Zambian preterm birth prevention study

## References

[1] UN Joint Programme on HIV/AIDS (UNAIDS) (2014). The gap report.

[2] Wedi COO, Kirtley S, Hopewell S, Corrigan R, Kennedy SH, Hemelaar J (2016). Perinatal outcomes associated with maternal HIV infection: A systematic review and meta-analysis. Lancet HIV.

[3] Obimbo MM, Zhou Y, Mcmaster MT, Cohen CR, Qureshi Z, Ong’ech J, et al. Placental structure in preterm birth amongst HIV positive versus negative women in Kenya. JAIDS J Acquir Immune Defic Syndr. 2018;1. doi:10.1097/QAI.0000000000001871. 10.1097/QAI.0000000000001871 PMC6289800 30272633

[4] Kalk E, Schubert P, Bettinger JA, Cotton MF, Esser M, Slogrove A (2017). Placental pathology in HIV infection at term: a comparison with HIV-uninfected women. Trop Med Int Heal.

[5] Conroy AL, McDonald CR, Gamble JL, Olwoch P, Natureeba P, Cohan D (2017). Altered angiogenesis as a common mechanism underlying preterm birth, small for gestational age, and stillbirth in women living with HIV. Am J Obstet Gynecol.

[6] Govender N, Naicker T, Moodley J (2013). Maternal imbalance between pro-angiogenic and anti-angiogenic factors in HIV-infected women with pre-eclampsia. Cardiovasc J Afr.

[7] Demir R, Seval Y, Huppertz B (2007). Vasculogenesis and angiogenesis in the early human placenta. Acta Histochem.

[8] Tian H, Huang JJ, Golzio C, Gao X, Hector-Greene M, Katsanis N (2018). Endoglin interacts with VEGFR2 to promote angiogenesis. FASEB J.

[9] Kaitu’u-Lino TJ, Whitehead CL, Ngian GL, Permezel M, Tong S. Serum concentrations of soluble flt-1 are decreased among women with a viable fetus and no symptoms of miscarriage destined for pregnancy loss. PLoS One. 2012;7(2):e32509. 10.1371/journal.pone.0032509 PMC3289655 22389705

[10] Jayasena CN, Abbara A, Comninos AN, Narayanaswamy S, Maffe JG, Izzi-Engbeaya C (2016). Novel circulating placental markers prokineticin-1, soluble fms-like tyrosine kinase-1, soluble endoglin and placental growth factor and association with late miscarriage. Hum Reprod.

[11] Daponte A, Pournaras S, Polyzos NP, Tsezou A, Skentou H, Anastasiadou F (2011). Soluble fms-like tyrosine kinase-1 (sFlt-1) and serum placental growth factor (PlGF) as biomarkers for ectopic pregnancy and missed abortion. J Clin Endocrinol Metab.

[12] Smith GCS, Crossley JA, Aitken DA, Jenkins N, Lyall F, Cameron AD (2007). Circulating angiogenic factors in early pregnancy and the risk of preeclampsia, intrauterine growth restriction, spontaneous preterm birth, and stillbirth. Obstet Gynecol.

[13] Asvold BO, Vatten LJ, Romundstad PR, Jenum PA, Karumanchi SA, Eskild A (2011). Angiogenic factors in maternal circulation and the risk of severe fetal growth restriction. Am J Epidemiol.

[14] Romero R, Chaiworapongsa T, Erez O, Tarca AL, Gervasi MT, Kusanovic JP (2010). An imbalance between angiogenic and anti-angiogenic factors precedes fetal death in a subset of patients: Results of a longitudinal study. J Matern Neonatal Med.

[15] Castillo MC, Fuseini NM, Rittenhouse K, Price JT, Freeman BL, Mwape H, et al. Zambian Preterm Birth Prevention Study (ZAPPS): cohort characteristics at enrollment. Gates Open Res. 2019;2:25. 10.12688/gatesopenres.12820.2 PMC6350406 30706053

[16] Price JT, Vwalika B, Rittenhouse KJ, Mwape H, Winston J, Freeman BL (2019). Adverse birth outcomes and their clinical phenotypes in an urban Zambian cohort. Gates Open Res.

[17] Palm M, Basu S, Larsson A, Wernroth L, Åkerud H, Axelsson O (2011). A longitudinal study of plasma levels of soluble fms-like tyrosine kinase 1 (sFlt1), placental growth factor (PlGF), sFlt1: PlGF ratio and vascular endothelial growth factor (VEGF-A) in normal pregnancy. Acta Obstet Gynecol Scand.

[18] Herraiz I, Simón E, Gómez-Arriaga PI, Martínez-Moratalla JM, García-Burguillo A, López Jiménez EA (2015). Angiogenesis-related biomarkers (sFlt-1/PLGF) in the prediction and diagnosis of placental dysfunction: An approach for clinical integration. Int J Mol Sci.

[19] Goldenberg RL, Gravett MG, Iams J, Papageorghiou AT, Waller SA, Kramer M (2012). The preterm birth syndrome: Issues to consider in creating a classification system. Am J Obstet Gynecol.

[20] Kramer MS, Papageorghiou A, Culhane J, Bhutta Z, Goldenberg RL, Gravett M (2012). Challenges in defining and classifying the preterm birth syndrome. Am J Obstet Gynecol.

[21] Papageorghiou AT, Kennedy SH, Salomon LJ, Altman DG, Ohuma EO, Stones W (2018). The INTERGROWTH-21st fetal growth standards: toward the global integration of pregnancy and pediatric care. Am J Obstet Gynecol.

[22] Govender L, Mackraj I, Gathiram P, Moodley J (2012). The role of angiogenic, anti-angiogenic and vasoactive factors in pre-eclamptic African women: Early- versus late-onset pre-eclampsia. Cardiovasc J Afr.

[23] Zera CA, Seely EW, Wilkins-Haug LE, Lim KH, Parry SI, McElrath TF (2014). The association of body mass index with serum angiogenic markers in normal and abnormal pregnancies. Am J Obstet Gynecol.

[24] Mayhew TM, Charnock-Jones DS, Kaufmann P (2004). Aspects of human fetoplacental vasculogenesis and angiogenesis III Changes in complicated pregnancies. Placenta.

[25] Chuchracki M, Krzyscin M, Dera-Szymanowska A, Pisarska M, Florek E, Breborowicz GH (2017). The role of sFLT-1 and PlGF factors as well as the sFLT-1/PlGF ratio as a predictor of gestational hypertension and preeclampsia. Arch Perinat Med.

[26] Price JT, Winston J, Vwalika B, Cole SR, Stoner MCD, Lubeya MK (2019). Quantifying bias between reported last menstrual period and ultrasonography estimates of gestational age in Lusaka. Zambia Int J Gynecol Obstet.

